# Brain Magnetic Resonance Imaging Classification Using Deep Learning Architectures with Gender and Age

**DOI:** 10.3390/s22051766

**Published:** 2022-02-24

**Authors:** Imayanmosha Wahlang, Arnab Kumar Maji, Goutam Saha, Prasun Chakrabarti, Michal Jasinski, Zbigniew Leonowicz, Elzbieta Jasinska

**Affiliations:** 1 Department of Information Technology, North-Eastern Hill University, Shillong 793022, India; imayanwahlang@gmail.com (I.W.); dr.goutamsaha@gmail.com (G.S.); 2 Techno India NJR Institute of Technology, Udaipur 313003, India; drprasun.cse@gmail.com; 3 Department of Electrical Engineering Fundamentals, Faculty of Electrical Engineering, Wroclaw University of Science and Technology, 50-370 Wroclaw, Poland; zbigniew.leonowicz@pwr.edu.pl; 4 Department of Operations Research and Business Intelligence, Wrocław University of Science and Technology, 50-370 Wrocław, Poland; elzbieta.jasinska@pwr.edu.pl

**Keywords:** brain tumor, Magnetic Resonance Imaging (MRI), deep learning, Convolutional Neural Network (CNN), Support Vector Machine (SVM), Deep Neural Network (DNN)

## Abstract

Usage of effective classification techniques on Magnetic Resonance Imaging (MRI) helps in the proper diagnosis of brain tumors. Previous studies have focused on the classification of normal (nontumorous) or abnormal (tumorous) brain MRIs using methods such as Support Vector Machine (SVM) and AlexNet. In this paper, deep learning architectures are used to classify brain MRI images into normal or abnormal. Gender and age are added as higher attributes for more accurate and meaningful classification. A deep learning Convolutional Neural Network (CNN)-based technique and a Deep Neural Network (DNN) are also proposed for effective classification. Other deep learning architectures such as LeNet, AlexNet, ResNet, and traditional approaches such as SVM are also implemented to analyze and compare the results. Age and gender biases are found to be more useful and play a key role in classification, and they can be considered essential factors in brain tumor analysis. It is also worth noting that, in most circumstances, the proposed technique outperforms both existing SVM and AlexNet. The overall accuracy obtained is 88% (LeNet Inspired Model) and 80% (CNN-DNN) compared to SVM (82%) and AlexNet (64%), with best accuracy of 100%, 92%, 92%, and 81%, respectively.

## 1. Introduction

The brain is the most complex organ present in the human body. It carries out different functions and controls the activities of other systems of the body. Additionally, the brain is comprised of complex structures including the cerebellum, cerebrum, and brain stem, which constitute the central nervous system [1,2]. The histology of the brain consists of brain cells and tissues. Brain cells are divided into neurons and neuroglia, and brain tissues into gray matter and white matter [2,3]. When cells of the brain grow abnormally and are not regulated correctly, it may result in a brain tumor. It is found that all variants of tumors are not cancerous. Fundamentally, cancer is a term used for malignant tumors, not benign tumors. Although benign tumors are less harmful than malignant tumors, the former still presents various problems in the brain [4]. There are many tests and medical imaging techniques that can be carried out for proper treatment. Some of the medical imaging techniques are Computed Tomography (CT), Magnetic Resonance Imaging (MRI), X-ray, etc. [5], but the standard way of evaluating a tumor is by using MRI due to its capability of achieving detailed images of the brain. A variety of brain conditions can be detected using MRI, including tumors, cysts, and other structural abnormalities. It can detect gray matter, white matter, and any damage or shunt present in the brain. Cerebrospinal fluids and the surrounding of tumors can be assessed by an MRI scan, which has a higher sensitivity for detecting the presence of a tumor. Detection of tumors at an early stage is essential, as it can be risky in many cases and can cause death in unfortunate circumstances. Therefore, prediction of the tumor using automated tools can be a great help in tumor identification and be the safest mode.

Detection of tumors can be accomplished by means of meticulous manual human analysis of MRI images one by one (slice by slice). This specific task needs to be performed for accurate identification of the region and the type of tumor. Additionally, tumors in the brain may affect certain other organs in a system (metastasis), which can be even more harmful. Detection of such tumors at an early stage is essential in selecting treatments in an efficient and effective decision-making capability on the part of the practitioner. Thus, proper analysis of brain MRI images is required to obtain valuable information which may be helpful in the early detection and diagnosis of diseases. In addition, early detection of tumors can lead to better diagnosis; to achieve this, the use of automated tools is the most reliable and aspiring contribution in medical science. Automated techniques have evolved in past decades in image processing, where traditional methods were used to solve such issues. This continues to shift towards more advanced techniques such as machine learning and eventually to deep learning, and other proposed methodologies [6].

**Figure 1:**
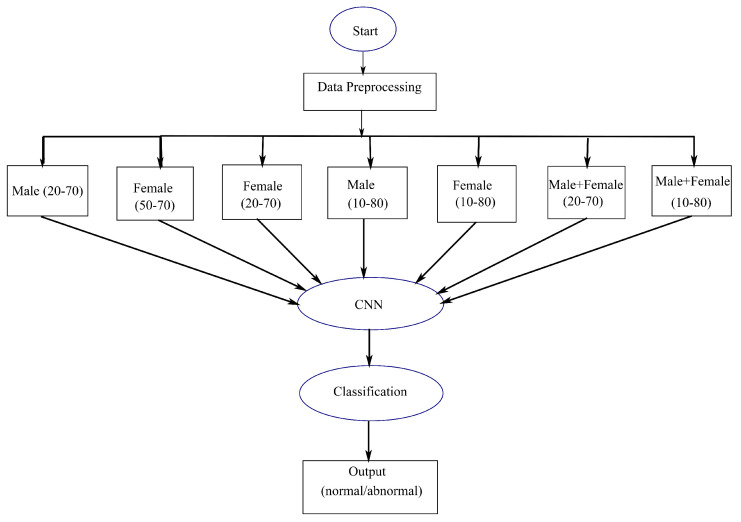
An overall flowchart, depicting proposed classification approach by using age and gender as attributes.

Keeping the necessity of manual examination, this paper includes state-of-the-art automated approaches to classify MRI images as normal (nontumorous) or abnormal (tumorous). For this purpose, a proposed deep learning based CNN methodology was used and compared with the existing techniques due to their superior performance in Computer Vision. We also divided the brain MRI images into different genders, male and female, and different age groups for classification into normal or abnormal. We incorporated age and gender as attributes for the first time, in contrast to earlier classification methodologies. This is crucial in determining similarities and differences of the brain concerning shape and size for different age groups and genders. This is in order to find out whether age and gender can be the factors in achieving a better result in classification; by finding similar patterns between images of the same category. A flowchart depicting the usages of age and gender bias (depending on data availability) is shown in Figure 1, where the data are taken and preprocessed using filtering and cropping. Based on available data obtained, the images are divided into seven categories based on different age groups and gender. These are then classified using proposed CNN models where output can be normal or abnormal. The following categories of brain MRI images were considered: (i) Males between the ages of 20 and 70, (ii) Females between the ages of 50 and 70, (iii) Females between the ages of 20 and 70, (iv) Males between the ages of 10 and 80, (v) Females between the ages of 10 and 80, (vi) Males and Females between the ages of 20 and 70, and (vii) Males and Females between the ages of 10 and 80. This is then applied to various approaches for classification as normal or abnormal.

### 1.1. Motivation

Previous research has focused on brain diagnosis as classified as either normal or abnormal. In earlier attempts, SVM has been utilized and achieved effective results in classification into normal or abnormal. Despite this, no higher attributes were used in its implementation. Though the accuracy of the existing approach is satisfactory with 99.9% accuracy, it may not be suitable for accurate prediction/classification of tumors, as human brain structure varies based on age and gender [7,8]. The information obtained using higher attributes is a reliable way to treat any kind of deformity. Such delicacy must be handled precisely for the proper diagnosis of diseases. Therefore, usage of higher attributes such as age, gender, etc., is much needed for accurate prediction, which leads to an appropriate diagnosis. In this paper, age and gender are taken as attributes for predicting the presence of tumors in the hope of obtaining an accurate result using CNN-based methodologies. In order to keep the network computationally cheaper, a deeper CNN is not used here, and higher depth may lead to poor generalization. In contrast to previous spatial exploitation-based CNNs such as AlexNet or VGGNets, a LeNet inspired model was chosen for its simplicity and use of a lower filter (3×3). This is more suited than other Nets due to less training time and is more computationally inexpensive.

### 1.2. Our Contributions

The main contributions of the paper are as follows:

1. Figshare [9], Brainweb [10], and Radiopaedia [11] datasets are readily available online and can be used to classify brain MRI as normal or abnormal. We have taken all these datasets to create a heterogeneous combination of data that address the heterogeneity issue. A dataset from the same source is used for the majority of studies in brain-related diagnosis. This form of heterogeneity has never been explored before, but it could be the beginning of correctly distinguishing images from different sources.
2. Using higher attributes is always more informative with a higher expectancy of reliable and efficient results. Here, work based on age and gender is considered as an initiative to determine whether these can be helpful in further automated diagnosis. It is inspired by the paper given in [12,13]. In addition to employing various data to classify patients as normal or abnormal, Radiopaedia datasets are used to classify patients by age and gender.
3. To categorize normal (absence of tumor) and abnormal (presence of tumor) images, two proposed CNN-based methodologies are applied. One is a model that is inspired from LeNet and the other is a Deep Neural Network based method. These proposed models are fast and more superficial compared to other comparable deep learning methods.
4. Two alternative deep learning-based classifiers, LeNet and ResNet, are incorporated in addition to the proposed methodology for classification. During their reign, these two models were used for classification and had a significant impact. They are utilized because they are not as deep as VGG19, MobileNet, Inception, and other state-of-the-art deep learning approaches, which are not ideal for our data as they are not massive and could lead to erroneous results and computational expense. To classify normal and abnormal images, the results are compared with Support Vector Machine and AlexNet, which were previously used to classify normal and abnormal images.
5. Compared to traditional SVM (82% using age and gender attributes and 77% using heterogenous data without any attributes), the parameters used in this paper are higher with better results and accuracy (88% using age and gender attributes and 80% using heterogenous data). While comparing to AlexNet, the depth and number of convolutions are lesser in the proposed method, making it simpler with more efficient computation time. AlexNet obtained an accuracy of 64% using age and gender attributes and 65% using heterogenous data without any attributes.
6. In this paper, data are not equally distributed for each group using age and gender. Data are unbalanced data, and cross-validation is used to solve this issue. This work is not clinically proven or tested, but it is performed to check the capability of a few deep-learning methodologies, mainly spatial CNN. This model might not work or perform well under different clinical settings, as data are obtained from online sources.

### 1.3. Organization of the Paper

This paper uses deep learning-based approaches to classify MRI images as normal or abnormal in a hope to see if using higher attributes can be beneficial. Section 2 includes works related to brain tumor classification and findings based on the anatomy of the brain of different individuals. Section 3 explains the types of methodologies used as well as the proposed method. Section 4 shows the result and findings, and in Section 5, the conclusion of the paper is given.

## 2. Related Works

Several existing works classify brain images into normal (tumorous) and abnormal (nontumorous). One such method can be seen in Rajesh et al. [14], where classification was implemented using Feed Forward Neural Network, consisting of three layers with 50 nodes in the hidden layer and one output node. Taie et al. [15] also performed the classification using Support Vector Machine (SVM), and comparative analysis can be seen in [16,17]. In another paper, Al-Baderneh et al. [18], also discussed the classification of brain MRI using Artificial Neural Network and K-Nearest Neighbor (KNN) with texture features, using 181 images of the abnormal brains and 94 images of normal brains. Other methodology includes Self Organizing Maps (SOM) which is discussed in [17,19]. Implementation of feedforward backpropagation for classification into normal or abnormal MRI images can be found in [20]. These methods are all supervised (classes are known), where features are needed to be extracted before classification. All of the above mentioned use traditional approaches with very few data with the efficient result but are not very informative and do not include age and gender bias.

Along with these methods, other state-of-the-art techniques using deep learning-based methodologies are evolving. Many of these works are not used to classify normal or abnormal but were included as the work was performed on brain imaging on different types of classification. In a paper by Pereira et al. [21] glioma detection was achieved using CNN. Kamnitsas et al. [22] used a deep learning method for the classification of ischemic stroke. In [23], a proposed method called Adaptive Network-based Fuzzy Inference System (ANFIS) for classification into five types of tumors was investigated. Another work focused on the classification and segmentation of tumors using pre-trained AlexNet, where features were extracted using the Gray-Level Co-Occurrence Matrix (GLCM) [24]. Other works include classification into different types of tumors using CNN [25,26,27,28,29], SVM [30], Graph cut [31], Recurrent Neural Network (RNN) [32,33], AlexNet transfer learning network of CNN [34], Deep Neural Network (DNN) [35,36,37], VGG-16, Inception V3 and ResNet50 [38], SVM and KNN [39], and CNN ensemble method [40].

In addition, other works include the MICCAI BRATS challenge; the most recent can be found in [41]. A comparative analysis of brain tumors can be seen in [42]. When it comes to differences in the human brain, an article by Brown [12] published studies on the human brain and differences in the structure of the brain and its morphology for individuals of the same age. Based on this, a model was developed using Pediatric Imaging, Neurocognition, and Genetics (PING) data to predict ages between 3 to 20 years old. It can also be seen that every individual brain measurement varies, even on a single brain at any specific time. This finding inspired us to investigate the brain structure further using an automated technique for identifying tumors according to gender and age. In the next section, we will discuss the different existing methods used for the classification of MRI into normal and abnormal.

### 2.1. A Brief Description on Existing Techniques Used in Classification of MRI into Normal and Abnormal

The most widely used machine learning algorithms for classification of brain MRI into normal and abnormal are Support Vector Machine (SVM) [15,16,17] and AlexNet [43]. A very brief description of each algorithm is presented in the next subsections.

#### 2.1.1. Support Vector Machine (SVM)

The most recent existing method, SVM is one of the most widely used supervised learning algorithms [15,16,17]. The advantages of using SVM are its memory efficiency and effectiveness in high dimensional spaces. It can also be used for regression. The SVM methodology was taken from [15]. The image was first converted into array. A label is assigned for all the images, 0 for normal class and 1 for abnormal class. Using SVM RBF kernel, an output of 0 or 1 is attained. The RBF kernel on two samples *X* and *X*′ is represented as

(1) $$K\left(X,X^{{}^{\prime }}\right)=exp\left(\frac{-(|X-X^{{}^{\prime }}{|}^{2})}{2\sigma^{2}}\right)$$

It is non parameterized, but using of $2\sigma^{2}$ makes it parameterized and it is known as Gaussian Radial Basis Function. It is commonly used as it is localized and it is a general purpose kernel used when no prior information is available about the data. The output obtained is 0 or 1, 0 for abnormal and 1 for normal class.

#### 2.1.2. AlexNet

AlexNet was designed by Alex Krizhevsky and is an award-winning architecture of ImageNet in 2012. It is a CNN based methodology that was originally used for classification of cats and dogs. The architecture can be seen in [43] consisting of five convolutional layers and three fully connected layers. A study which uses AlexNet as one of the steps in classification and segmentation of abnormalities can be seen in [24].

In this paper, we are going to classify the brain MRI images into normal or abnormal based on a specific range of ages, as it is already established by Brown [12] that the structure of the brain varies according to age. This will indeed help in finding a similar pattern of images of different ages. The main differences of our work from other existing works are the use of data from different sources and using age and gender as attributes in classification into normal or abnormal, which is the novelty of our work. Furthermore, compared to other works, our data usage is higher even though it is still considered a small dataset. Some comparisons based on related works are given in Table 1.

## 3. Classification of Brain MRI Images Using Deep Learning Architectures

**Figure 2:**
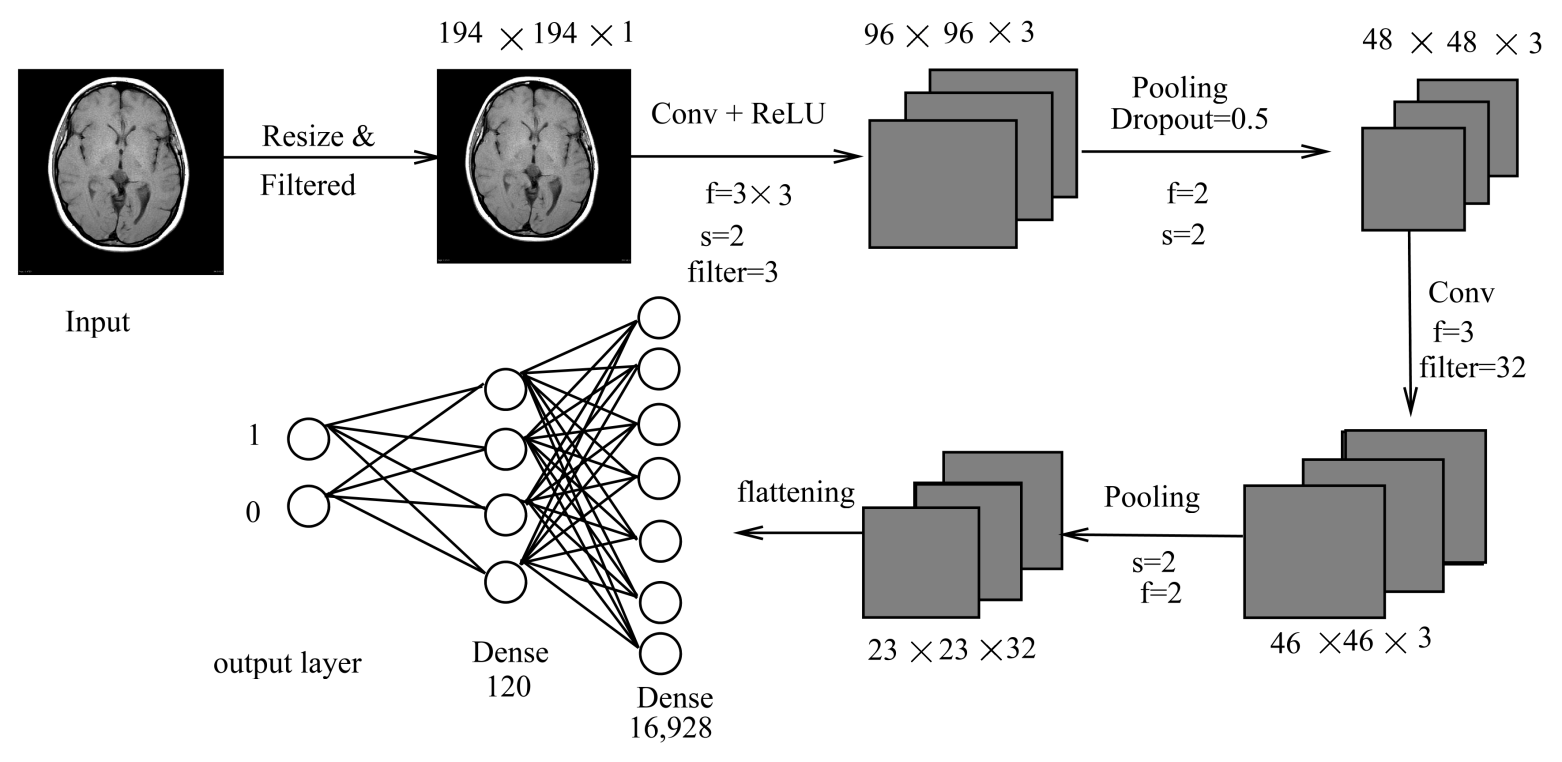
LeNet inspired model (LIM).

Classification plays a crucial role as it organizes images into specific groups. It is the initial step for predicting an area or region containing abnormalities in diagnosing any disease. In this section, along with the proposed methodology, three other deep learning architectures (LeNet, AlexNet, and ResNet50) are briefly discussed. The proposed classification technique for brain MRI images was performed using CNN due to its effective performance in image classification that automatically detects essential features. The brain images were classified into normal or abnormal classes, and the whole process is depicted in Figure 2. One method is a CNN-based approach with all the layers being used as per observations and formulation based on Equations (2) and (3). Using this method, classification was performed for different ages and genders to determine their similarities and differences. The imaging technique utilized here is MRI Fluid Attenuated Inversion Recovery (FLAIR) [44]. It is similar to a T2 image with a longer echo (TE) and relaxation time (TR). This sequence is very sensitive to pathology and makes the differentiation between Cerebrospinal Fluid (CSF) and an abnormality much easier [44].

### 3.1. Proposed Methodology

#### 3.1.1. LeNet Inspired Model

The proposed classification is a CNN-based model where the convolutional, pooling, and fully connected layers were used, as shown in Figure 2. It is inspired by LeNet architecture with minute changes, which is simple and has five layers (convolution and pooling layer). The input image (X) is in color format and has a size of N×N×3. Original images and augmented images are of different sizes. The images are cropped by selecting only the brain region. Our first step involves preprocessing to remove noises present in an image. It is carried out using median filtering. Median filtering is chosen to remove the outliers without affecting the information present in an image. After median filtering, the images are resized to a specific size of 194×194×1 to ensure the images are not too small; this is in order to maintain the ratio and helps in better training if sizes are all the same. The dimension of 194 is chosen as it is the smallest size of images available. The images are converted into a grayscale image for better learning of features. These images are then passed to the most important part of a CNN, which is the convolutional layer. In each convolutional layer, stride varies, as can be seen in Figure 2. Mathematically, inputs $X_{1},X_{2},\cdots X_{N}$ with size N×N, using f×f filters will give an output of $\sum_{i=1}^{N}X_{i}^{l}\times W_{i}^{l}$ where $W_{i}$ is the window of the filter and output size can be obtained using $\frac{N+2p-f}{s}+1\times \frac{N+2p-f}{s}+1$ (*f* is the filter, *p* is the padding, and *s* is the stride; *p* and *s* ≥ 0, *f* > 1).

(2) $$W_{l+1}=\frac{W_{l}-f}{s}+1$$

(3) $$Y_{i,j,d}=max\left\{0,X_{i,j,d}^{l}\right\}$$

where $0\leq i<H^{l}=H^{l+1}$, $0\leq j<W^{l}=W^{l+1}$, and $0\leq d<D^{l}=D^{l+1}$ where *H* is the height, *W* is width, and *D* is the depth of an image. As there is no parameter inside ReLU, no parameter is learned during this layer. A stride of 2×2 is used which moves two positions of pixels vertically and horizontally. At each stride, a maximum of four numbers are taken and replaced by a single value. For example, for a 94×94×16 input size, an output of 46×46×16 is obtained, whereas a stride of 1 will not reduce much in size. Filter size was taken as 3×3 for local features learning and not a bigger filter size such as 11×11. Depth of 12 and 16, respectively, was chosen arbitrarily for deeper depth, as our image has a depth of 1. As our dataset in not that huge, convolution is taken as per our requirements with total of two convolutional layers. After every layer, the image is shrunk and edge information may be reduced. This is reduced using padding. In our work, no padding is applied as reduction is still needed until the last convolutional layer. Max pooling is applied for reduction in sizes with stride of 2×2. After the last convolutional layer, a fully connected layer is followed with a total of 23×23×32= 16,928 number of neurons, which are then passed to another fully connected layer of size 800. Optimization was not performed using Gradient descent (GD) but using Adam optimizer (adaptive moment estimation). It is similar to GD, but it has an advantage over it as it maintains learning rate for each weight in a network. Dropout, which is a regularizer, is used in fully connected layers in our method. The rate of 0.5 is given for this purpose. A loss function that is used was binary cross-entropy loss function (log loss) [45]. It can be calculated using:

(4) $$H_{p}\left(q\right)=-\frac{1}{N}\sum_{i=1}^{N}y_{i}\cdot log\left(P\left(y_{i}\right)\right)+\left(1-y_{i}\right)\cdot log\left(1-p\left(y_{i}\right)\right)$$

where *y* is the label (1 for class 1 and 0 for class 2), p(y) is the probability of being a class 1 for all N inputs, and $p(y_{i})$ is the predicted probability for all *N* samples given any distribution *q*(*y*). Probability of each point is $\frac{1}{N}$. For each *y* = 1, it adds log(*p*(*y*)), the probability of being in class 1 and for *y* = 0, log(1−p(*y*)) the probability of being in class 2. This gives a better loss in comparison with any other loss in all cases. Lastly, with Adam optimizer, Softmax is used for classification where value <0.5 is classified into [1 0] (abnormal) otherwise [0 1] (normal).

#### 3.1.2. CNN Combined with DNN (CNN-DNN)

**Figure 3:**
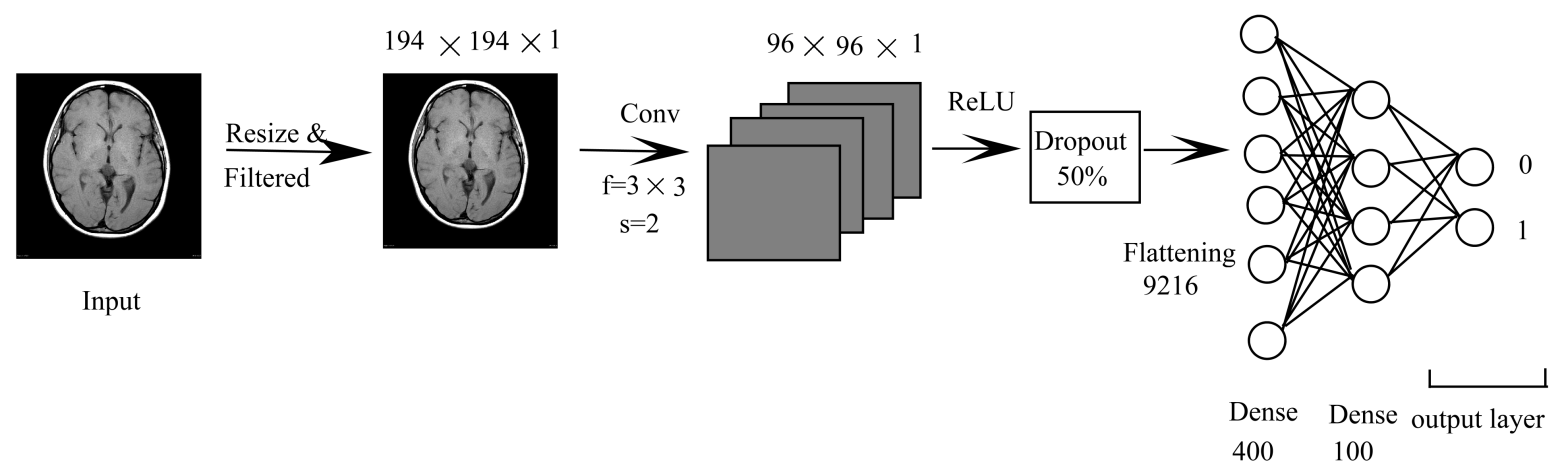
CNN-DNN.

This method has been taken due to the simple approach, and it is not so widely used but applicable in many fields of computer vision. The diagram showing CNN-DNN is shown in Figure 3. The network starts with the input image being passed to a convolutional layer with a filter size of 3×3 stride of 2×2 after resizing into 194×194. Then, it is passed to a ReLU layer with the dropout rate of 50%, which is then passed to a fully connected layer with 962,312 nodes. It is then followed by a dense layer of 400 and 100 and a classification layer that classifies into 0 or 1 using a Softmax classifier.

Other than the proposed architectures, we have also implemented a few known deep learning architectures for effective comparison, which are provided next.

### 3.2. LeNet

LeNet is one of the most widely used and popular network architectures in deep learning. This model is popularly implemented for the classification of objects in different domains of computer vision and hand written text using MNIST dataset. The reason for this is its simplicity and smaller number of layers. The architecture with the same parameters are used with some minor changes. The changes made were based on batch size, loss function, and the number of epoch. The architecture of LeNet can be seen in [46].

### 3.3. ResNet50 (Transfer Learning)

ResNet won first place on the ILSVRC 2015 classification task using ImageNet data. The architecture can be seen in [47]. For this work, ResNet50, depth based CNN, is used as a model for transfer learning. Transfer learning is flexible where the pre-trained model is used directly for classifying images. The architecture stays the same with a flatten layer and two additional dense layers. Using the dataset considered for our work, the model is trained and modified into two-class problems where the output is class 0 (abnormal) and 1 (normal).

The parameters used are changed according to our dataset, and the same number of epoch is taken for all the cases, which is 100 as output converges at this point. The differences in parameters between our method and the others can be seen in Table 2.

A comparison can be made based on computational complexity. The computational complexity (*CC*) of a convolutional network is measured in terms of the total number of learnable parameters [48]. It can be expressed as:

(5) CC=2cwh(X−w+1)(Y−h+1)

where *X* and *Y* are the height and width of the input image, respectively; *w* and *h* are the width and height of the convolution kernel, respectively; and *c* is the number of channels.

Using this, ResNet has the highest computational complexity, and it is more time consuming compared to any other methods used. In this work, based on computational complexity, the Nets can be ranked as AlexNet>ResNet>CNN−DNN>LIM>LeNet where trainable parameters values are approximately 30 million (M), 23 M, 3 M, 3 M, and 2 M, respectively.

## 4. Experimental Results

A Python programming language is used to carry out the implementation. We are using a web application Google Colab, which is an open-source application. Libraries used are Keras and TensorFlow. SVM, LIM, CNN-DNN, LeNet, AlexNet, and ResNet50 are implemented to classify the images as normal or abnormal. The implementation is carried out in two parts; firstly, generalized classification into normal or abnormal without using age and gender, and secondly, classification into normal or abnormal using age range and gender. Two approaches are used, firstly, k fold cross-validation with k fold = 5 and 8 (arbitrarily chosen), and secondly, generalization approach, where the data in the training phase are not used in the testing phase.

### 4.1. Performance Metrics

Many performance metrics are considered by researchers in classification, based on which Accuracy is the popularly used performance metric. For checking the validity of our result, the parameters used are Accuracy, Precision, Sensitivity, Specificity, Negative Predictive Value, False Positive Rate, False Discovery Rate, False Negative Rate, F1 Score, Matthews Correlation Coefficient, and Loss Function [49]. The different performance metrics with their description are provided in Table 3.

### 4.2. Normal or Abnormal Classification

T1 weighted and FLAIR data were used in this work, collected from Figshare, Brainweb, and Radiopaedia. A total of 1130 images were used in Figshare, which contains abnormal data. Each slice of T1 weighted data in Brainweb contains 181 slices of normal and abnormal data. Cropping was used to increase the number of slices, resulting in 362 slices per image. In addition, 768 T1 images and FLAIR data were taken from Radiopaedia. For this case, no data augmentation has been used. For k fold cross-validation, there are 2530 images, with 806 and 1534 normal and abnormal images, respectively. A total of 506 images are utilized for testing purposes using the generalization approach. The output obtained using k fold cross-validation and a generalization method for LeNet, AlexNet, ResNet, SVM, LIM, and CNN-DNN is given in Table 4.

**Figure 4:**
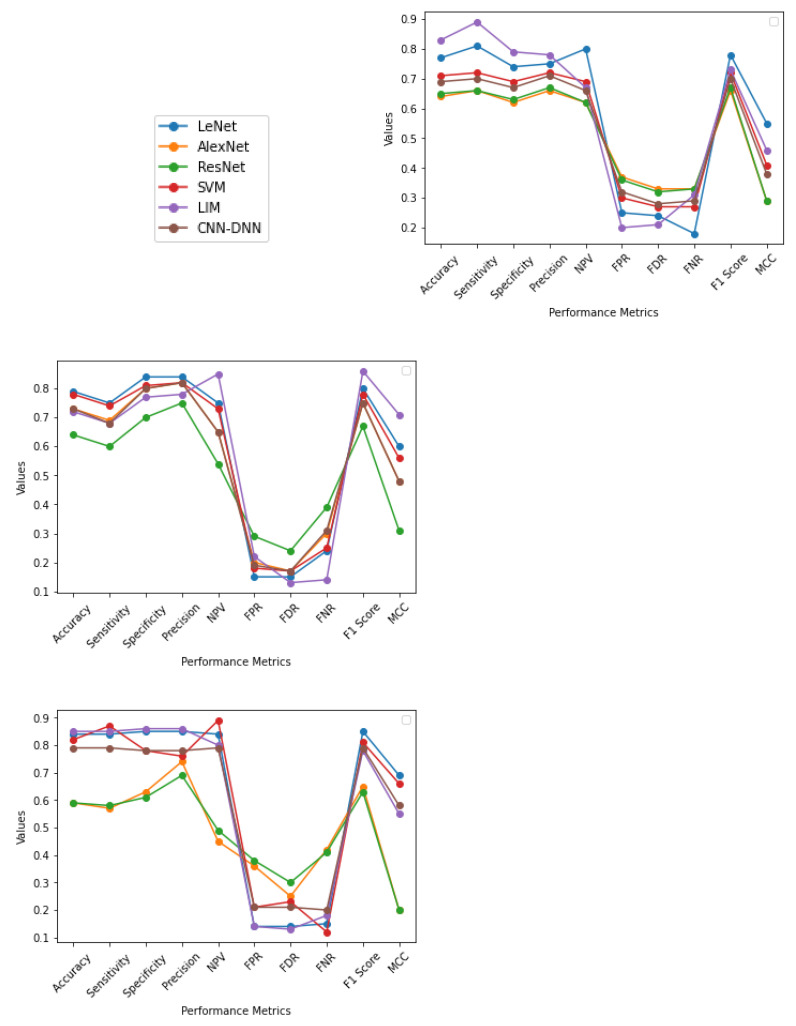
The graphs illustrate the Accuracy, Specificity, Sensitivity, Precision, Recall, F1 Score, NPV, FPR, FDR, FNR, and MCC of AlexNet, ResNet, SVM, LeNet, LIM, and CNN-DNN for five-fold, eight-fold, and generalization approach, respectively, with values ranging from −1 to 1.

From the output shown in Table 4 and Figure 4 it is observed that, for five-fold cross-validation, Accuracy, Specificity, Sensitivity, Precision, FPR, FDR, FNR, F1 score, and MCC are better in the case of LIM, and NPV in the case of SVM. For an eight-fold comparison, LeNet has better Accuracy, Specificity, Sensitivity, and Precision, whereas LIM has better NPV, FPR, FDR, FNR, F1 score, and MCC. In generalization approach Accuracy, Specificity, Sensitivity, Precision, and FDR are better in LIM; NPV and FNR in SVM; and FPR, F1 score, and MCC, are better in LeNet; in SVM, the Accuracy attained is relatively low in some circumstances due to data heterogeneity. In most cases, employing a cross-fold validation and generalization approach, LIM and LeNet produce better results than SVM methodology. It is also worth noting that less dense Nets provide higher True Positive values than a denser network such as ResNet.

### 4.3. Range Based Classification

For both normal (nontumorous) and abnormal (tumorous) images, the data were collected from Radiopaedia [11]. The images obtained were not all from the same patient, ensuring that distinct tumors were present. The images were divided into several age groups to perform experiments based on male or female gender or both. The ranges are not sequentially ordered and are repeated when data for a specific age are not available or when there are not any data at all. In order to identify these images and conduct the experiment, it was assumed that the data gathered came from the same MRI scan.

Based on their ages and gender, the images were divided into distinct ranges. This aids in the identification of essential and robust logical conclusions about brain size similarities across different ranges: Male (20–70), Male (10–80), Female (50–70), Female (20–70), Female (10–80), Male and Female (20–70), Male and Female (10–80), Male and Female (20–70), and Male and Female (10–80). Due to the lower number of images used, these were all cropped for data augmentation. There are 1205 images, 786 of which are abnormal and 411 of which are normal. The generalization approach uses 328 images from the aggregate data for testing purposes. It becomes much more manageable by dividing it into ranges, and it confirms that age and gender as attributes can be used to detect similarities and classify into normal or abnormal class.

**Figure 5:**
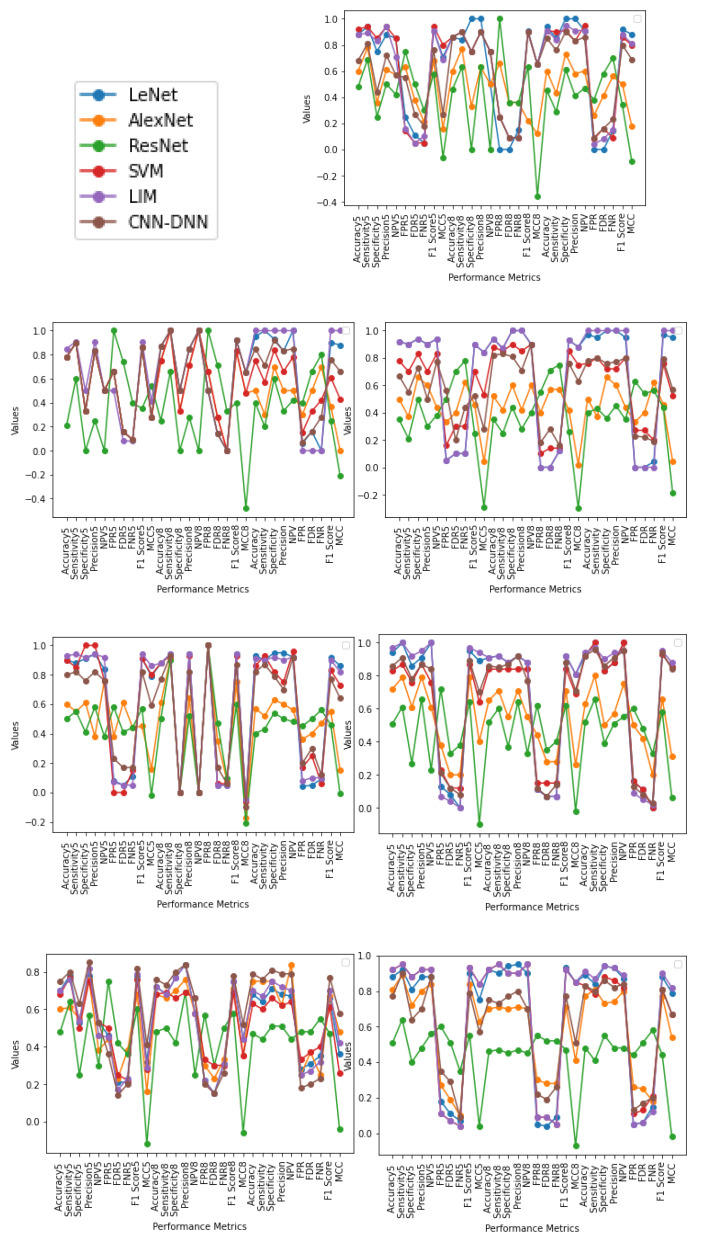
The graphs illustrate the Accuracy, Specificity, Sensitivity, Precision, Recall, F1 Score, NPV, FPR, FDR, FNR, and MCC of LeNet, AlexNet, ResNet, SVM, LIM, and CNN-DNN for Male (20–70), Female (50–70), Female (20–70), Male (10–80), Female (10–80), Male + Female (10-80), and Male + Female (10–80), respectively. Represented as five-fold with ending 5, eight-fold with ending 8, and generalization approach for each performance metrics with values ranging from −1 to 1.

From Figure 5 and output obtained in Table 5, Table 6, Table 7, Table 8, Table 9 and Table 10, it can be seen that, for Male (20–70) using five-fold cross-validation, SVM gives a better result compared to LIM; LIM and LeNet gives a second-best result, in the case of eight-fold cross-validation, LeNet provides a better result compared to LIM and SVM which gives second best; and using generalization approach LeNet gives a better result, followed by LIM. For Female (50–70) using five-fold cross-validation, LIM gives the best result compared to all other methods; in the case of eight-fold cross-validation, LeNet and LIM provide a better result compared to CNN-DNN and SVM; and using generalization method, LIM gives a better result compared to LeNet, SVM, and CNN-DNN. For Female (20–70), using five-fold cross-validation LIM and LeNet give a better result compared to other methods. In the case of eight-fold cross validation, LIM provides a better result, and using the generalization method, LIM gives a better result. For Male (10–80), using five-fold cross validation, LIM gives a better result compared to other methods; in the case of eight-fold cross validation, LIM and LeNet provide better results; and using the generalization method, LIM gives a better result. For Male (10–80), using five-fold cross-validation LIM gives a better result compared to other methods; in the case of eight-fold cross validation, LeNet provides better results; and using the generalization method, LeNet gives a better result, followed by LIM. For Female (10–80), using five-fold cross-validation LIM gives a better result compared to other methods; in the case of eight-fold cross validation, LeNet and LIM provide better results; and using the generalization method, LIM gives a better result, followed by LeNet and then by SVM. For Male + Female (20–70) using five-fold cross-validation, LIM gives a better result compared to other methods; in the case of eight-fold cross-validation, CNN-DNN provides better result, followed by LeNet and LIM; and using generalization method, CNN-DNN gives a better result, followed by LIM. For Male + Female (10–50) using five-fold cross-validation, LIM and SVM give a better result compared to other methods; in the case of eight-fold cross-validation, LIM and SVM provide better results, followed by LeNet and LIM; and using generalization method, LIM gives a better result compared to other methods.

### 4.4. Statistical Significance Test

The T-test and Analysis of Variance (ANOVA) test are two often-used statistical tests [50]. Statistical tests show the significance of the model. Here, we have performed the ANOVA test using Python programming library for statistical test (scipy.stats). From Table 11, for classification into normal or abnormal, both the models are significant, as the *p*-value is less than the significance level (0.05). There is a statistical improvement using LIM and CNN-DNN over SVM, AlexNet, and ResNet, but no improvement over LeNet. In the case of classification using gender and age, there seems to be a false discovery rate producing conflicting results. LIM shows a significant difference over other models considering majority cases, both values in green and bold, with no improvement over LeNet. The test indicates that the proposed LIM can be considered equal to LeNet and outperforms SVM. There is a difference between the groups, considering deeper networks such as AlexNet and ResNet feature in both classifications with different variance and are statistically significant.

It can be observed that the result using both males and females is more distinguishable, and males or females of all ranges as separate inputs show statistical significance difference, wherein we can say that age is a more dominating factor than gender. However, it is not enough to conclude if any individual variable is significant from our output. The *p*-value using the ANOVA test for samples between two models is high in age and gender classification into normal or abnormal because samples have a value of 0 and 1 with fewer testing samples, unlike classification without using age and gender having heterogeneous data with more testing samples.

### 4.5. Benefits and Drawbacks of Our Methods

The benefits of the proposed methodologies are their simplicity and fast implementation. Though they are not as deep as other Nets available, they are still comparable to LeNet and other basic CNNs. They are spatial exploitation-based approach CNNs, with fewer layers, less training time, and less computational expense. The main aim of these methods is to find the applicability of CNN in classification into normal or abnormal classes in the simplest form. Dropout is used for overfitting purposes, similar to that of AlexNet with ReLU and Softmax activation functions. This model has no advanced structures such as residual networks, pathways, or deep and dense networks. It is as simple as LeNet and AlexNet, with computational complexity in between the two.

Although this method proves to be equivalent to other machine learning approaches, this method might not perform well when the data used are different under different settings and different datasets. This work uses unbalanced data, which can also be different from using balanced data. It is a quest in determining the capability of using deep learning models that are not deeper or wider. This model is not dense enough, which is another drawback. Additionally, this work is technical, not clinical, and not under the supervision of an expert but based on the datasets provided on the websites. The data used were from freely available online data.

A brief discussion and interpretation of comparison between the five methodologies is given in the next section.

### 4.6. Summary

The following findings and discussion can be concluded based on the experimental results:

1. Using age and gender as attributes with a range of ages is more informative, as it involves higher attributes and, as a result, is less biased. This helps in effective and efficient analysis of the brain and its abnormalities.
2. In most instances, classification into normal or abnormal without using age and gender as attributes yields less accurate results. This shows that using age and gender attributes is relevant and valuable in the classification of brains into normal or abnormal class.
3. The pattern obtained in the case of Female (20–70) and Male + Female (10–80) yielded better results than that of other age range in almost all methodologies which signifies that using age and gender as attributes are essential and can help in better classification of a tumor. Furthermore, the same applies in the case of Male + Female, where age acts as a significant factor in providing an efficient and reliable classification where, taking gender as a factor, the result is accurate in most cases.
4. This can be interpreted as though the output is better differentiated when both male and female are taken as separate inputs. It can be observed that assumptions of the same age range of the same gender are likely to have similar patterns, as output is better in most cases. This is because brain volume varies by 50% even in the group of the same age and varies differently for different genders [7,8]. Gender as a factor has shown a more promising result.
5. From performance metrics and ANOVA tests, using gender can be considered a relevant factor as the pattern and output are better when taking Male or Female as a separate input; also, when combining the gender of all ages, the pattern does not change much, which can imply that gender is a dominating factor over age. The pattern obtained in the case of Male (10–80) and Female (10–80) does not provide a better result than when combining the two genders in all methodologies (except in a few cases using statistical test), which shows that similarities between males and females could be differentiated better using gender as an attribute. Using both age and gender attributes thus acts as an essential factor in providing better accuracy in diagnosis as a whole.
6. In most cases, the output is better when CNN-based methodologies are applied instead of the SVM method. In several cases, LIM is in first or second place. On the other hand, CNN-DNN can be comparable to SVM in output provided by the generalization and k fold cross-validation approaches. This shows that deep learning methodologies have the potential to achieve reliable results through further experiments in the future. The deep learning model has more layers and provides finer details at a deeper level about the images, which act as a tool for a better prognosis.
7. Although gender is more dominating than age as per our utilized data and result, it is not enough to say whether any variable is statistically significant based on the ANOVA test. On the other hand, the model (LIM) is statistically significant. Using higher variables as a relevant factor is reasonable based on performance metrics and the ANOVA test.

## 5. Conclusions and Future Work

Finding a treatment for various types of brain tumors has become one of the most important areas of medical imaging. Considering Accuracy, Specificity, Sensitivity, Precision, Recall, F1 Score, NPV, FPR, FDR, FNR, and MCC, LIM performs better in this paper for the first case. In most cases, employing a cross-fold validation and generalization strategy, LIM and CNN-DNN produce better results than SVM and AlexNet when dealing with heterogeneous data. LIM follows a similar pattern to the original LeNet, but it is unable to overcome it. In the second case, it was discovered that brain classification works better for brains of different ages and genders than for the brains of the same gender using LIM, CNN-DNN, and the other four methodologies. It is due to the similarities patterns between the same genders. In other words, it can be concluded that the pattern and characteristic features of the same gender are likely to be similar. Additionally, from statistical tests and performance metrics, gender can be considered a factor in the future analysis of the brain, with age as a factor as well. The accuracy is not high due to the presence of noise and heterogeneity in the data, where the methods could not differentiate between normal and abnormal images properly. An overall Accuracy using age and gender as attributes of SVM, AlexNet, ResNet, LeNet, LIM, and CNN-DNN is 82%, 64%, 44%, 87%, 88%, and 80%, respectively, and best accuracy of 92%, 81%, 52%, 97%, 100%, and 92%, respectively. Deeper networks, such as AlexNet and ResNet, were unable to produce the desired results due to their capacity for handling large amounts of data, which was limited in our case, and different setting. In addition, the data used in our case are unbalanced data which usually provide lower accuracy compared to using balanced data. Using gender as a factor, the result was more promising and is a reasonably good factor to be taken into consideration in the automated diagnosis of the brain. Overall, both age and gender are significant factors for obtaining effective and efficient results. Classifying normal or abnormal brain MRI data will be more informative and accurate with age as an attribute.

The application of deep learning-based methodologies such as CNN outperforms traditional methods, including SVM, which has the highest classification accuracy to date. More tests on brain size may be performed using large amounts of data, taking gender and suitable age range as attributes, as this can be used to reach a higher level of accuracy than a generalized classification. Classification and segmentation-based works are engaging; however, a more efficient method is needed for these purposes. Researchers are still looking for a way to reduce human effort and make the processes of detecting brain tumors and other abnormalities more efficient. Deep learning has the potential to tackle and provide higher accuracy, dependability, and efficiency.

## Figures and Tables

**Table 1:** Comparison of existing methodologies.

Paper and Year	Method	Classification	Dataset Used	Accuracy (%)
Al-Baderneh et al. (2012) [18]	NN and KNN	Normal/Abnormal	275 images	100 and 98.92
Rajesh et al. (2013) [14]	Feed Forward Neural Network	Normal/Abnormal	20 images	90
Taie et al. (2017) [15]	SVM		80, 100, and 150 images	90.89 and 100
krishnammal et al. (2019) [24]	AlexNet	Benign/Malignant	Not mention	100
Hanwat et al. (2019) [25]	CNN	Benign/Malignant/ Normal	94 images	71
Hamid et al. (2020) [30]	DWT, GLM, and SVM	Benign/Malignant	Dicom images	95
Kulkarni et al. (2020) [34]	AlexNet	Benign/Malignant	75 Benign and 75 Malignant images	98.44 (F measure)

**Table 2:** Parameter differences and number of layers used in proposed method, LeNet, AlexNet and ResNet.

Parameter Name	LeNet	AlexNet	ResNet	LIM	CNN-DNN
Number of convolution layer	2	5	48	2	1
Number of pooling layer	2 (2×2)	3 (2×2)	24 (2×2)	2 (2×2)	Nil
Depth	32	96	512	32	3
Filter size	5×5	11×11, 3×3, 5×5	3×3, 7×7	3×3	3×3
Loss function	binary crossentropy	binary crossentropy	binary crossentropy	binary crossentropy	binary crossentropy
Classifier	Sigmoid	Softmax	Softmax	Softmax	Sigmoid
Number of Dropout	3	3	10	2	2
Dropout rate	0.5	0.5	0.5	0.5	0.5
Activation Function	tanH	ReLU	ReLU	ReLU	Sigmoid
Optimizer	Sgd	Sgd	Adam	Adam	Adam
Model type	cascade	cascade	cascade	cascade	cascade

**Table 3:** Performance metrics used.

No.	Performance Metric	Description
1	Accuracy	Accuracy is a measurement that gives the correctness of classification and loss is a measure indicating that how well a model behaves after every iteration.
2	Precision	The fraction of true positives (TP) from the total amount of relevant result. Precision = TP/(TP + FP).
3	Recall (Sensitivity)	The fraction of true positives from the total amount of TP and FN. Recall = TP/(TP + FN).
4	F1 Score	The harmonic mean of Precision and Recall given by the following formula: F1 = 2 ∗ (TP ∗ FP)/(TP + FP)
5	Specificity	Specificity = TN/(FP + TN)
6	Negative Predictive Value	NPV = TN/(TN + FN)
7	False Positive Rate	FPR = FP/(FP + TN)
8	False Discovery Rate	FDR = FP (FP + TP)
9	False Negative Rate	FNR = FN/(FN + TP)
10	Matthews Correlation Coefficient	TP ∗ TN − FP ∗ FN/sqrt((TP + FP) ∗ (TP + FN) ∗ (TN + FP) ∗ (TN + FN))

**Table 4:** Output obtained for LeNet, AlexNet, ResNet, SVM, LIM, and CNN-DNN for classification into normal or abnormal with best result highlighted in bold.

Methods	Phase	Parameters	Five-Fold	Eight-Fold	Generalization	Methods	Phase	Parameters	5 Fold	Eight-Fold	Generalization
		Accuracy	0.79	**0.82**	0.83			Accuracy	0.80	0.81	0.83
	Training	Loss	0.44	0.37	0.42		Training	Loss	NA	NA	NA
		Accuracy	0.77	**0.79**	0.84			Accuracy	0.71	0.78	0.82
		Sensitivity	0.81	**0.75**	0.84			Sensitivity	0.72	0.74	0.87
		Specificity	0.74	**0.84**	0.85			Specificity	0.69	0.81	0.78
		Precision	0.75	**0.84**	0.85			Precision	0.72	0.82	0.76
		NPV	0.80	0.75	0.84			NPV	**0.69**	0.73	**0.89**
		FPR	0.25	0.15	**0.14**			FPR	0.30	0.18	0.21
		FDR	0.24	0.15	0.14			FDR	0.27	0.17	0.23
		FNR	0.18	0.24	0.15			FNR	0.27	0.25	**0.12**
		F1 Score	0.78	0.80	**0.85**			F1 Score	0.72	0.78	0.81
		MCC	0.55	0.60	**0.69**			MCC	0.41	0.56	0.66
LeNet	Testing	Loss	0.42	**0.43**	0.40	SVM	Testing	Loss	NA	NA	NA
		Accuracy	0.97	0.73	0.55			Accuracy	**0.81**	0.68	**0.90**
	Training	Loss	**0.07**	0.77	5.54		Training	Loss	0.41	**0.36**	**0.20**
		Accuracy	0.64	0.73	0.59			Accuracy	**0.83**	0.72	**0.85**
		Sensitivity	0.66	0.69	0.57			Sensitivity	**0.89**	0.68	**0.85**
		Specificity	0.62	0.80	0.63			Specificity	**0.79**	0.77	**0.86**
		Precision	0.66	0.82	0.74			Precision	**0.78**	0.78	**0.86**
		NPV	0.62	0.65	0.45			NPV	0.67	**0.85**	0.80
		FPR	0.37	0.20	0.36			FPR	**0.20**	**0.22**	0.14
		FDR	0.33	0.17	0.25			FDR	**0.21**	**0.13**	**0.13**
		FNR	0.33	0.30	0.42			FNR	**0.31**	**0.14**	0.18
		F1 Score	0.66	0.75	0.65			F1 Score	**0.73**	**0.86**	0.78
		MCC	0.29	0.48	0.20			MCC	**0.46**	**0.71**	0.55
AlexNet	Testing	Loss	1.33	0.95	5.94	LIM	Testing	Loss	**0.39**	0.60	**0.39**
		Accuracy	0.67	0.70	0.65			Accuracy	0.81	0.80	0.81
	Training	Loss	0.70	0.76	0.83		Training	Loss	0.49	0.50	0.55
		Accuracy	0.65	0.64	0.59			Accuracy	0.69	0.73	0.79
		Sensitivity	0.66	0.60	0.58			Sensitivity	0.70	0.68	0.79
		Specificity	0.63	0.70	0.61			Specificity	0.67	0.80	0.78
		Precision	0.67	0.75	0.69			Precision	0.71	0.82	0.78
		NPV	0.62	0.54	0.49			NPV	0.66	0.65	0.79
		FPR	0.36	0.29	0.38			FPR	0.32	0.19	0.21
		FDR	0.32	0.24	0.30			FDR	0.28	0.17	0.21
		FNR	0.33	0.39	0.41			FNR	0.29	0.31	0.20
		F1 Score	0.67	0.67	0.63			F1 Score	0.70	0.75	0.79
		MCC	0.29	0.31	0.20			MCC	0.38	0.48	0.58
ResNet	Testing	Loss	0.74	0.64	0.83	CNNDNN	Testing	Loss	0.55	0.56	0.61

**Table 5:** LeNet output using age and gender (Gen = Generalization approach).

Age and Gender	Approach	Training		Testing										
		**Accuracy**	**Loss**	**Accuracy**	**Sensitivity**	**Specificity**	**Precision**	**NPV**	**FPR**	**FDR**	**FNR**	**F1 Score**	**MCC**	**Loss**
Male (20–70)	Five-fold	0.93	0.28	0.88	0.94	0.75	0.88	0.85	0.25	0.11	0.05	0.91	0.71	0.38
	Eight-fold	0.95	0.72	0.86	0.84	1	1	0.5	0	0	0.15	0.91	0.65	0.43
	Gen	0.93	0.12	0.94	0.85	1	1	0.91	0	0	0.14	0.92	0.88	0.10
Female (50–70)	Five-fold	0.92	0.41	0.78	0.90	0.33	0.83	0.50	0.66	0.16	0.09	0.86	0.28	0.43
	Eight-fold	1	0.12	0.87	1	0.50	0.85	1	0.50	0.14	0	0.92	0.65	0.48
	Gen	1	0.09	0.95	1	0.93	0.83	1	0.06	0.16	0	0.90	0.88	0.09
Female (20–70)	Five-fold	0.96	0.15	0.92	0.90	0.94	0.90	0.94	0.05	0.1	0.1	0.90	0.84	0.14
	Eight-fold	0.96	0.14	0.94	0.87	1	1	0.90	0	0	0.12	0.93	0.88	0.15
	Gen	0.92	0.19	0.97	0.95	1	1	0.95	0	0	0.04	0.97	0.95	0.15
Male (10–80)	Five-fold	0.89	0.37	0.90	0.88	0.91	0.94	0.84	0.08	0.05	0.11	0.91	0.79	0.43
	Eight-fold	0.88	0.27	0.88	0.94	0	0.94	0	1	0.05	0.05	0.94	−0.05	0.21
	Gen	0.88	0.20	0.93	0.90	0.95	0.95	0.92	0.04	0.05	0.09	0.92	0.86	0.17
Female (10–80)	Five-fold	0.94	0.21	0.94	1	0.86	0.91	1	0.13	0.08	0	0.95	0.89	0.20
	Eight-fold	1	0.09	0.91	0.92	0.88	0.92	0.88	0.11	0.07	0.07	0.92	0.81	0.18
	Gen	0.95	0.14	0.92	1	0.83	0.88	1	0.16	0.11	0	0.93	0.85	0.14
Male + Female (20–70)	Five-fold	0.70	0.57	0.70	0.78	0.53	0.78	0.53	0.46	0.21	0.21	0.78	0.32	0.52
	Eight-fold	0.76	0.51	0.72	0.68	0.77	0.84	0.58	0.22	0.15	0.31	0.75	0.44	0.55
	Gen	0.76	0.31	0.68	0.64	0.71	0.68	0.67	0.28	0.31	0.35	0.66	0.36	0.37
Male + Female (10–80)	Five-fold	0.93	0.32	0.88	0.92	0.81	0.88	0.88	0.18	0.11	0.07	0.90	0.75	0.26
	Eight-fold	0.96	0.16	0.92	0.90	0.94	0.95	0.90	0.05	0.04	0.09	0.93	0.85	0.23
	Gen	0.91	0.19	0.89	0.84	0.94	0.93	0.87	0.05	0.06	0.15	0.88	0.79	0.19

**Table 6:** AlexNet output using age and gender (Gen = Generalization approach).

Age and Gender	Approach	Training		Testing										
		Accuracy	Loss	Accuracy	Sensitivity	Specificity	Precision	NPV	FPR	FDR	FNR	F1 Score	MCC	Loss
Male (20–70)	Five-fold	0.60	1.37	0.60	0.78	0.36	0.61	0.57	0.63	0.38	0.21	0.68	0.16	1.61
	Eight-fold	0.83	0.67	0.60	0.77	0.33	0.63	0.50	0.66	0.36	0.36	0.22	0.12	1.51
	Gen	0.72	0.90	0.60	0.43	0.73	0.58	0.60	0.26	0.41	0.56	0.50	0.18	1.58
Female (50–70)	Five-fold	0.76	1.04	0.78	0.90	0.33	0.83	0.50	0.66	0.16	0.09	0.86	0.28	0.99
	Eight-fold	0.62	1.72	0.75	1	0.33	0.71	1	0.66	0.28	0	0.83	0.48	0.96
	Gen	0.78	1.02	0.50	0.30	0.70	0.50	0.50	0.30	0.50	0.70	0.37	0	0.98
Female (20–70)	Five-fold	0.46	1.11	0.50	0.37	0.66	0.60	0.44	0.33	0.40	0.62	0.46	0.04	0.93
	Eight-fold	0.88	0.24	0.52	0.42	0.60	0.42	0.60	0.40	0.57	0.57	0.42	0.02	0.84
	Gen	0.56	0.84	0.50	0.42	0.47	0.59	0.40	0.52	0.40	0.48	0.55	−0.00	0.94
Male (10–80)	Five-fold	0.68	0.57	0.60	0.55	0.61	0.38	0.76	0.38	0.61	0.44	0.45	0.16	0.79
	Eight-fold	0.61	1.0	0.61	0.91	0	0.64	0	1	0.35	0.08	0.75	−0.17	0.84
	Gen	0.68	1.04	0.57	0.52	0.63	0.60	0.56	0.36	0.40	0.47	0.55	0.15	1.78
Female (10–80)	Five-fold	0.91	0.51	0.72	0.79	0.61	0.79	0.61	0.38	0.20	0.20	0.79	0.40	0.55
	Eight-fold	0.50	0.82	0.65	0.71	0.55	0.71	0.55	0.44	0.28	0.28	0.71	0.26	1.15
	Gen	0.68	0.76	0.63	0.80	0.50	0.57	0.75	0.50	0.42	0.20	0.66	0.31	0.81
Male + Female (20–70)	Five-fold	0.65	1.11	0.60	0.61	0.55	0.76	0.38	0.44	0.23	0.38	0.68	0.16	1.16
	Eight-fold	0.68	0.96	0.68	0.66	0.70	0.76	0.58	0.30	0.23	0.33	0.71	0.35	0.90
	Gen	0.80	0.75	0.75	0.75	0.75	0.62	0.84	0.25	0.37	0.25	0.67	0.48	1.18
Male + Female (10–80)	Five-fold	0.61	1.48	0.81	0.89	0.72	0.80	0.84	0.27	0.19	0.10	0.84	0.63	0.87
	Eight-fold	0.81	0.77	0.70	0.71	0.70	0.71	0.70	0.30	0.28	0.28	0.71	0.41	0.94
	Gen	0.81	0.52	0.77	0.81	0.73	0.74	0.80	0.26	0.25	0.18	0.77	0.54	0.62

**Table 7:** ResNet output using age and gender (Gen = Generalization approach).

Age and Gender	Approach	Training		Testing										
		Accuracy	Loss	Accuracy	Sensitivity	Specificity	Precision	NPV	FPR	FDR	FNR	F1 Score	MCC	Loss
Male (20–70)	Five-fold	0.54	0.69	0.48	0.69	0.25	0.50	0.42	0.75	0.50	0.30	0.58	−0.06	0.73
	Eight-fold	0.65	0.82	0.46	0.63	0	0.63	0	1	0.36	0.36	0.63	−0.36	0.74
	Gen	0.45	0.70	0.45	0.29	0.61	0.41	0.47	0.38	0.58	0.70	0.34	−0.09	0.74
Female (50–70)	Five-fold	0.15	1.20	0.21	0.60	0	0.25	0	1	0.74	0.40	0.35	0.54	0.86
	Eight-fold	0.23	0.83	0.25	0.66	0	0.28	0	1	0.71	0.33	0.40	−0.48	0.79
	Gen	0.61	0.68	0.40	0.20	0.60	0.33	0.42	0.40	0.66	0.80	0.25	−0.21	0.79
Female (20–70)	Five-fold	0.21	0.71	0.35	0.21	0.50	0.30	0.38	0.50	0.70	0.78	0.25	−0.29	0.79
	Eight-fold	0.46	0.69	0.35	0.25	0.44	0.28	0.40	0.55	0.71	0.75	0.26	−0.30	0.75
	Gen	0.39	0.90	0.40	0.43	0.36	0.45	0.35	0.63	0.54	0.56	0.44	−0.19	0.87
Male (10–80)	Five-fold	0.48	0.73	0.50	0.55	0.41	0.58	0.38	0.58	0.41	0.44	0.57	−0.02	0.77
	Eight-fold	0.73	0.57	0.50	0.90	0	0.52	0	1	0.47	0.10	0.60	−0.21	0.73
	Gen	0.34	0.81	0.48	0.43	0.54	0.50	0.48	0.45	0.50	0.56	0.46	−0.01	0.64
Female (10–80)	Five-fold	0.61	0.68	0.51	0.61	0.27	0.66	0.23	0.72	0.33	0.38	0.64	−0.10	0.68
	Eight-fold	0.55	0.68	0.52	0.60	0.37	0.64	0.33	0.62	0.35	0.40	0.62	−0.02	0.69
	Gen	0.48	0.68	0.52	0.66	0.39	0.51	0.55	0.60	0.48	0.33	0.58	0.06	0.61
Male + Female (20–70)	Five-fold	0.53	0.75	0.48	0.64	0.25	0.57	0.30	0.75	0.42	0.36	0.60	−0.12	0.86
	Eight-fold	0.59	0.71	0.48	0.50	0.42	0.69	0.25	0.57	0.30	0.50	0.58	−0.06	0.78
	Gen	0.47	0.96	0.47	0.44	0.51	0.51	0.44	0.48	0.48	0.55	0.47	−0.04	0.95
Male + Female (10–80)	Five-fold	0.50	0.78	0.51	0.64	0.40	0.48	0.56	0.60	0.51	0.35	0.55	0.04	0.76
	Eight-fold	0.69	0.68	0.46	0.47	0.45	0.47	0.45	0.55	0.52	0.52	0.47	−0.07	0.85
	Gen	0.39	0.88	0.48	0.41	0.55	0.48	0.48	0.44	0.51	0.58	0.44	−0.02	0.80

**Table 8:** SVM output using age and gender (Gen = Generalization approach).

Age and Gender	Approach	Training		Testing										
			Accuracy	Loss	Accuracy	Sensitivity	Specificity	Precision	NPV	FPR	FDR	FNR	F1 Score	MCC	Loss
Male (20–70)	Five-fold	0.91	NA	0.92	0.94	0.85	0.94	0.85	0.14	0.05	0.05	0.94	0.80	NA
	Eight-fold	0.97	NA	0.86	0.90	0.75	0.90	0.75	0.25	0.09	0.09	0.90	0.65	NA
	Gen	0.91	NA	0.91	0.90	0.91	0.83	0.95	0.08	0.16	0.09	0.86	0.80	NA
Female (50–70)	Five-fold	0.96	NA	0.78	0.90	0.33	0.83	0.50	0.66	0.16	0.09	0.86	0.28	NA
	Eight-fold	0.96	NA	0.75	1	0.33	0.71	1	0.66	0.28	0	0.83	0.48	NA
	Gen	0.76	NA	0.75	0.57	0.84	0.66	0.78	0.15	0.33	0.42	0.61	0.43	NA
Female (20–70)	Five-fold	0.99	NA	0.78	0.70	0.83	0.70	0.83	0.16	0.30	0.30	0.70	0.53	NA
	Eight-fold	0.95	NA	0.88	0.85	0.90	0.85	0.90	0.10	0.14	0.14	0.85	0.75	NA
	Gen	0.80	NA	0.76	0.80	0.72	0.72	0.80	0.27	0.27	0.20	0.76	0.52	NA
Male (10–80)	Five-fold	0.93	NA	0.90	0.85	1	1	0.76	0	0	0.15	0.91	0.80	NA
	Eight-fold	0.92	NA	0.88	0.93	0	0.93	0	1	0.06	0.06	0.93	−0.06	NA
	Gen	0.86	NA	0.86	0.93	0.82	0.75	0.96	0.17	0.25	0.06	0.83	0.73	NA
Female (10–80)	Five-fold	0.97	NA	0.83	0.87	0.76	0.87	0.76	0.23	0.12	0.12	0.87	0.64	0.20
	Eight-fold	0.97	NA	0.84	0.84	0.84	0.84	0.84	0.15	0.15	0.15	0.84	0.69	NA
	Gen	0.91	NA	0.92	1	0.83	0.88	1	0.16	0.11	0	0.93	0.85	NA
Male + Female (20–70)	Five-fold	0.69	NA	0.68	0.77	0.50	0.75	0.53	0.50	0.25	0.22	0.76	0.28	NA
	Eight-fold	0.71	NA	0.68	0.69	0.66	0.69	0.66	0.33	0.30	0.30	0.69	0.35	NA
	Gen	0.62	NA	0.63	0.60	0.66	0.62	0.64	0.33	0.37	0.40	0.61	0.26	NA
Male + Female (10–80)	Five-fold	0.95	NA	0.92	0.95	0.88	0.92	0.92	0.11	0.07	0.04	0.93	0.84	NA
	Eight-fold	0.95	NA	0.92	0.95	0.90	0.90	0.95	0.09	0.09	0.05	0.92	0.85	NA
	Gen	0.90	NA	0.83	0.78	0.88	0.86	0.82	0.11	0.13	0.21	0.81	0.67	NA

**Table 9:** LIM using age and gender (Gen = Generalization approach).

Age and Gender	Approach	Training		Testing										
			Accuracy	Loss	Accuracy	Sensitivity	Specificity	Precision	NPV	FPR	FDR	FNR	F1 Score	MCC	Loss
Male (20–70)	Five-fold	0.92	0.20	0.88	0.89	0.83	0.94	0.71	0.16	0.05	0.10	0.91	0.69	0.29
	Eight-fold	0.93	0.16	0.86	0.90	0.75	0.90	0.75	0.25	0.09	0.09	0.90	0.65	0.13
	Gen	0.91	0.51	0.91	0.84	0.95	0.91	0.91	0.04	0.08	0.15	0.88	0.81	0.50
Female (50–70)	Five-fold	0.93	0.20	0.85	0.91	0.50	0.91	0.50	0.50	0.08	0.08	0.91	0.41	0.34
	Eight-fold	1	0.15	0.87	1	0.50	0.85	1	0.50	0.14	0	0.92	0.65	0.29
	Gen	1	0.11	1	1	1	1	1	0	0	0	1	1	0.11
Female (20–70)	Five-fold	1	0.06	0.92	0.90	0.94	0.90	0.94	0.05	0.1	0.1	0.90	0.84	0.12
	Eight-fold	1	0.22	0.94	0.87	1	1	0.90	0	0	0.12	0.93	0.88	0.27
	Gen	0.92	0.17	1	1	1	1	1	0	0	0	1	1	0.10
Male (10–80)	Five-fold	0.93	0.29	0.93	0.94	0.92	0.94	0.92	0.07	0.05	0.05	0.94	0.86	0.29
	Eight-fold	0.94	0.24	0.88	0.94	0	0.94	0	1	0.05	0.05	0.94	−0.05	0.31
	Gen	0.89	0.29	0.91	0.90	0.92	0.90	0.92	0.08	0.10	0.10	0.90	0.82	0.29
Female (10–80)	Five-fold	0.97	0.18	0.97	1	0.92	0.95	1	0.07	0.04	0	0.97	0.94	0.12
	Eight-fold	1	0.09	0.91	0.92	0.88	0.92	0.88	0.11	0.07	0.07	0.92	0.81	0.16
	Gen	1	0.12	0.94	0.97	0.90	0.94	0.95	0.09	0.05	0.02	0.95	0.88	0.17
Male + Female (20–70)	Five-fold	0.73	0.44	0.70	0.76	0.54	0.82	0.46	0.45	0.17	0.23	0.79	0.29	0.46
	Eight-fold	0.78	0.52	0.72	0.68	0.77	0.84	0.58	0.22	0.15	0.31	0.75	0.44	0.51
	Gen	0.73	0.62	0.70	0.67	0.75	0.72	0.70	0.25	0.27	0.32	0.70	0.42	0.50
Male + Female (10–80)	Five-fold	0.97	0.17	0.92	0.95	0.88	0.92	0.92	0.11	0.07	0.04	0.93	0.84	0.22
	Eight-fold	1	0.11	0.92	0.95	0.90	0.90	0.95	0.09	0.09	0.05	0.92	0.85	0.20
	Gen	0.92	0.29	0.91	0.87	0.94	0.93	0.89	0.05	0.06	0.12	0.90	0.82	0.24

**Table 10:** CNN-DNN using age and gender (Gen = Generalization approach).

Age and Gender	Approach	Training		Testing										
		Accuracy	Loss	Accuracy	Sensitivity	Specificity	Precision	NPV	FPR	FDR	FNR	F1 Score	MCC	Loss
Male (20–70)	Five-fold	0.73	0.66	0.68	0.81	0.44	0.72	0.57	0.55	0.27	0.18	0.76	0.27	0.35
	Eight-fold	0.58	0.69	0.86	0.90	0.75	0.90	0.75	0.25	0.09	0.09	0.90	0.65	0.63
	Gen	0.75	0.50	0.85	0.76	0.90	0.83	0.86	0.09	0.16	0.23	0.80	0.69	0.46
Female (50–70)	Five-fold	0.84	0.42	0.78	0.90	0.33	0.83	0.50	0.66	0.16	0.09	0.86	0.28	0.44
	Eight-fold	0.87	0.40	0.87	1	0.50	0.85	1	0.50	0.14	0	0.92	0.65	0.43
	Gen	0.87	0.27	0.85	0.71	0.92	0.83	0.85	0.07	0.16	0.28	0.76	0.66	0.27
Female (20–70)	Five-fold	0.75	0.43	0.67	0.55	0.73	0.50	0.77	0.56	0.20	0.44	0.52	0.28	0.51
	Eight-fold	1	0.16	0.82	0.83	0.81	0.71	0.90	0.18	0.28	0.16	0.76	0.63	0.37
	Gen	0.89	0.59	0.78	0.80	0.76	0.77	0.80	0.23	0.22	0.19	0.79	0.57	0.70
Male (10–80)	Five-fold	0.88	0.27	0.80	0.82	0.76	0.82	0.76	0.23	0.17	0.17	0.82	0.59	0.40
	Eight-fold	0.81	0.29	0.77	0.93	0	0.82	0	1	0.17	0.06	0.87	−0.10	0.36
	Gen	0.86	0.28	0.82	0.87	0.79	0.70	0.92	0.20	0.30	0.12	0.77	0.64	0.20
Female (10–80)	Five-fold	0.51	0.78	0.86	0.91	0.78	0.87	0.84	0.21	0.12	0.08	0.89	0.70	0.58
	Eight-fold	0.88	0.39	0.86	0.85	0.87	0.92	0.77	0.12	0.07	0.14	0.88	0.71	0.47
	Gen	0.83	0.43	0.92	0.96	0.86	0.91	0.95	0.13	0.08	0.03	0.94	0.84	0.37
Male + Female (20–70)	Five-fold	0.70	0.58	0.75	0.80	0.63	0.85	0.53	0.36	0.14	0.20	0.82	0.41	0.45
	Eight-fold	0.84	0.41	0.76	0.73	0.80	0.84	0.66	0.20	0.15	0.26	0.78	0.52	0.49
	Gen	0.88	0.40	0.79	0.76	0.81	0.79	0.79	0.18	0.20	0.23	0.77	0.58	0.36
Male + Female (10–80)	Five-fold	0.81	0.41	0.77	0.90	0.64	0.70	0.88	0.35	0.29	0.09	0.79	0.57	0.47
	Eight-fold	0.88	0.42	0.75	0.73	0.77	0.80	0.70	0.22	0.19	0.26	0.77	0.51	0.49
	Gen	0.81	0.41	0.83	0.80	0.86	0.82	0.84	0.13	0.17	0.20	0.81	0.67	0.37

**Table 11:** Statistical test (ANOVA) of LIM and CNN-DNN with respect to SVM, LeNet, AlexNet, and ResNet where values marked as ** are *p*-values < 0.05 and * are *p*-values < 0.1.

	Categories	LIM vs. SVM	LIM vs. AlexNet	LIM vs. LeNet	LIM vs. ResNet	CNN-DNN vs. SVM	CNN-DNN vs. AlexNet	CNN-DNN vs. LeNet	CNN-DNN vs. ResNet
**Normal/Abnormal** **Classification**	**Generalization**	** 0.03	** 1.48 × 10${}^{-3}$	0.94	** 0.02	**0.07**	** 2.33 × 10${}^{-6}$	0.70	** 0.0009
Range Based Classification	Male (20–70)	0.24	* 0.06	0.34	** 0.04	0.24	* 0.06	0.34	** 0.04
	Female (50–70)	0.10	* 0.06	0.11	* 0.06	1	0.35	0.33	0.17
	Female (20–70)	0.35	** 0.04	0.1	** 0.04	0.21	* 0.09	0.18	0.18
	Male (10–80)	** 0.02	* 0.06	0.76	** 0.04	1	** 0.03	0.13	** 0.03
	Female (10–80)	* 0.08	* 0.08	* 0.08	** 0.04	0.14	* 0.08	0.14	** 0.03
	Male + Female (20–70)	** 0.03	** 0.02	1	** 0.02	1	** 0.03	0.28	** 0.03
	Male + Female (10–80)	**0.02	* 0.08	0.86	** 0.02	0.33	0.13	0.71	0.46

## Data Availability

The data is obtained from Figshare Available online: https://figshare.com/, BrainWeb: Simulated Brain Database. Available online: https://brainweb.bic.mni.mcgill.ca/brainweb/ and Radiopaedia. Available online: https://radiopaedia.org/cases.
